# Impact of Dietary Gluten on Regulatory T Cells and Th17 Cells in BALB/c Mice

**DOI:** 10.1371/journal.pone.0033315

**Published:** 2012-03-13

**Authors:** Julie Christine Antvorskov, Petra Fundova, Karsten Buschard, David P. Funda

**Affiliations:** 1 The Bartholin Institute, Rigshospitalet, Copenhagen, Denmark; 2 Department of Immunology and Gnotobiology, Institute of Microbiology, v.v.i., Czech Academy of Sciences, Prague, Czech Republic; La Jolla Institute for Allergy and Immunology, United States of America

## Abstract

Dietary gluten influences the development of type 1 diabetes (T1D) and a gluten-free (GF) diet has a protective effect on the development of T1D. Gluten may influence T1D due to its direct effect on intestinal immunity; however, these mechanisms have not been adequately studied. We studied the effect of a GF diet compared to a gluten-containing standard (STD) diet on selected T cell subsets, associated with regulatory functions as well as proinflammatory Th17 cells, in BALB/c mice. Furthermore, we assessed diet-induced changes in the expression of various T cell markers, and determined if changes were confined to intestinal or non-intestinal lymphoid compartments. The gluten-containing STD diet led to a significantly decreased proportion of γδ T cells in all lymphoid compartments studied, although an increase was detected in some γδ T cell subsets (CD8^+^, CD103^+^). Further, it decreased the proportion of CD4^+^CD62L^+^ T cells in Peyer's patches. Interestingly, no diet-induced changes were found among CD4^+^Foxp3^+^ T cells or CD3^+^CD49b^+^cells (NKT cells) and CD3^−^CD49b^+^ (NK) cells. Mice fed the STD diet showed increased proportions of CD4^+^CD45RB^high+^ and CD103^+^ T cells and a lower proportion of CD4^+^CD45RB^low+^ T cells in both mucosal and non-mucosal compartments. The Th17 cell population, associated with the development of autoimmunity, was substantially increased in pancreatic lymph nodes of mice fed the STD diet. Collectively, our data indicate that dietary gluten influences multiple regulatory T cell subsets as well as Th17 cells in mucosal lymphoid tissue while fewer differences were observed in non-mucosal lymphoid compartments.

## Introduction

Several studies in non-obese diabetic (NOD) mice as well as Biobreeding (BB) rats have documented that the pathogenesis of type 1 diabetes (T1D) is influenced by diet [1]. It has been demonstrated that a gluten-free (GF) diet largely prevented diabetes onset in NOD mice: the diabetes incidence was reduced from 64% to 15% [2], and a cereal-based diet promotes diabetes development [3]. Furthermore, two large human prospective cohort studies have established a connection between early infant diet containing gluten and the development of autoantibodies against the pancreatic islets. Both studies found an increased risk (≥4) of islet autoimmunity when children were exposed to gluten-containing cereals early in life [4], [5]. Moreover, studies have documented an association between T1D and celiac disease (CD), which is a disease with several autoimmune features in which gluten is the triggering agent in genetic predisposed individuals [6]. It has been proposed that undiagnosed CD increases the risk of developing secondary autoimmune disorders including T1D [7]. The prevalence of CD in children with T1D has been reported to be 2–12%, and patients with CD have an earlier onset of diabetes compared to T1D patients without CD [8], [9]. Thus, dietary gluten seems to be an etiological or disease-influencing factor in T1D.

Changes in intestinal permeability have been described both in spontaneous animal models of T1D [10] and in human disease [11], [12]. Changes in permeability might be a direct effect of the gliadin fraction of gluten-containing cereals, because gliadin increases zonulin release, which opens intestinal tight junctions [13], [14]. There is also evidence for a primary role of the intestinal immune system in the pathogenesis of T1D. Diabetogenic T cells are initially primed in the gut [15], islet-infiltrating T cells express gut-associated homing receptors [16] and mesenteric lymphocytes transfer diabetes from NOD-mice to NOD/scid-mice [17].

The role of the intestinal immune system in the pathogenesis of T1D is important, because the gut is the physiological induction site of protective immunity and is a barrier to the outer environment. Proper development of mucosal immune responses is required for induction of tolerance vs. inflammation, controlled by various subsets of T cells and dendritic cells (DC). In both human and mice, several different T cell subsets with regulatory properties (Tregs) have been showed to play a role in maintaining a tolerant state and prevent autoimmune reactions [18].

In the present study we compared the effect of a diabetes-protective GF diet to a diabetes-permissive gluten-containing STD diet, on proportions of selected T cell subsets associated with regulatory functions (γδ T cells, NKT cells and Foxp3^+^ T cells), as well as NK cells and proinflammatory Th17 cells, in fully immunocompetent BALB/c mice. Furthermore, we studied diet-induced changes in the expression of different T cell markers (CD103, CD45RB^high/low^ and CD62L) and determined if these changes were located within mucosal lymphoid tissues (Peyer's patches (PP), mesenteric (MLN), pancreatic (PLN) lymph nodes) or the non-mucosal lymphoid compartments (spleen (S), inguinal (ILN) lymph nodes).

## Materials and Methods

### Animals

Timed pregnant BALB/cA BomTac mice were purchased from Taconic Europe A/S, Ejby, Denmark and kept in a Specific Pathogen Free (SPF) animal facility at the Panum Institute, Copenhagen (temperature 22±2 degrees, 12 h light cycle, air changed 16 times pr hour, humidity 55±10%) with free access to water and food. At day seven after birth, female pups and the female parent were assigned randomly into two groups, to receive either the STD, gluten-containing or the gluten-free (GF) diet. Twelve (six in each group) first generation female offspring were used in the study when 6 weeks old. The experiments were performed in two independent times. The animal experiments were carried out with approval from The National Animal Experimentation Board, and experiments were performed in accordance with international guidelines for the care and use of laboratory animals.

### Diets

The animals received either the STD, non-purified Altromin 1310 diet, or a GF, modified Altromin diet (Altromin, Lage, Germany). Both experimental diets were nutritionally adequate with a similar level of protein, amino acids, minerals, vitamins and trace element, only the protein source differed between the diets. These two diets have been previously used at The Bartholin Institute, to study the effect of a GF diet on diabetes incidence in NOD mice [2], [19]. The exact composition of the STD and the GF diet is given in [2], [19]. The protein content of the GF diet and the STD were similar (22.7% vs. 22.9%). Proteins in the STD diet were derived from wheat (25%), maize, and soya, whereas the GF diet protein source was meat and soya proteins. The two diets also had the same content of amino acids, minerals, vitamins and trace elements. The weight of the mice was monitored and both groups of animals displayed similar weight distribution.

### Antibodies

The following monoclonal antibodies (mAb) were purchased from BD Pharmingen: FITC-conjugated rat anti-mouse CD45RB mAb (16A; IgG2a, κ); FITC-conjugated rat anti-mouse CD49b mAb (DX5;IgM; κ); PE-conjugated rat anti-mouse CD25 mAb (3C7; IgG2b), PE-conjugated rat anti-mouse CD62L mAb (MEL-14; IgG2a, κ); PE-conjugated rat anti-mouse CD4 mAb (H129.19; IgG2a, κ), PerCP 5.5-conjugated hamster anti-mouse CD3 mAb (145-2C11; IgG1, κ), FITC-conjugated rat anti-mouse CD103 mAb (M290;IgG2a, κ), PE-conjugated hamster anti-mouse γδ T-cell receptor mAb (GL3; IgG2, κ), PerCP-Cy5.5 rat anti-mouse CD8a mAb (53-6.7; IgG2a, κ). Purchased from eBioscience were: PE-conjugated rat anti-mouse Foxp3 mAb (FJK-16s; IgG2a, κ), FITC-conjugated rat anti-mouse CD4 mAb (RM4-5; IgG2a, κ), Alexa Flour 488-conjugated rat anti-mouse IL-17A mAb (eBio17B7; IgG2a, κ).

### Cell purification and flow cytometry

Mice were sacrificed and S, ILN, MLN, PP, PLN were isolated. Cells from each organ were pooled and single-cell suspensions were prepared. Surface staining was initiated with use of the relevant mAb, and cells were incubated for 1/2 hour. Intracellular staining was carried out using the Mouse Regulatory T Cell Staining Kit (eBiocience FJK-16s, Cat.No. 88-8111) following the manufacturer procedure. Fc block (CD16/CD32) was purchased from BD Pharmingen (2.4G2; IgG2b, κ) and added to reduce Fc receptor-mediated binding. The cells were analysed by flow cytometry using a FACSscan (BD Bioscience), and data were analysed with use of CellQuest software (BD Bioscience). Isotype control antibodies were used to determine the amount of non-specific binding, and propidium iodide was used to exclude or to localize dead cells.

### Statistical analysis

Student's unpaired *t*–test was used to compare the frequency of the cells (means ± SEM) of the two groups (STD vs. GF) of mice, and a value of P<0.05 was considered statistically significant. Statistical analysis and graphic presentation of the results were performed using the Sigma Plot 9.0 Software and GraphPad Prism version 5.

## Results

### No diet-induced difference was found in the proportion of CD4^+^Foxp3^+^ T cells

**Figure 1:**
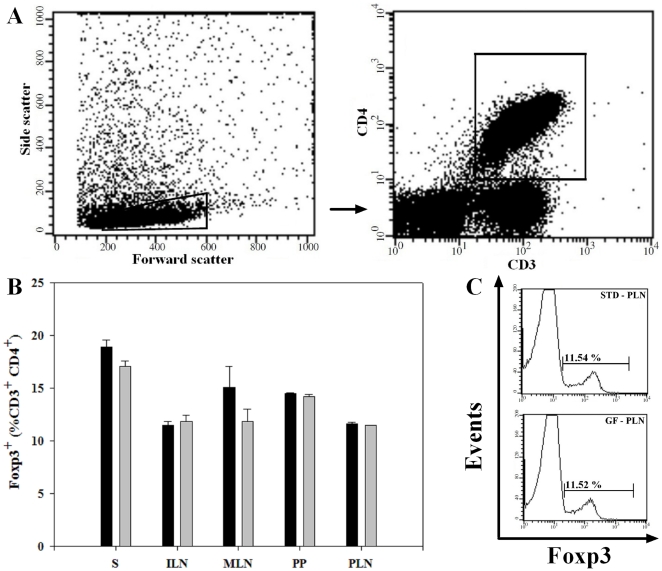
No diet-induced difference was found in the proportion of CD4^+^Foxp3^+^ T cells. (**A**) Representative plot of lymphocyte and CD3^+^CD4^+^ gate. (**B**) Bars represent the proportion of Foxp3^+^ cells (gated on 100% CD4^+^CD3^+^ cells) in various lymphoid compartments. No diet-induced differences was found in the proportion of CD4^+^Foxp3^+^ T cells in all lymphoid compartments tested. Data are presented as mean values ± SEM of two independent experiments with 6 mice in each group. (**C**) Representative histogram from PLN, showing the proportion of CD4^+^Foxp3^+^T cells in the STD diet mice compared to the GF diet mice. Spleen (S); inguinal lymph nodes (ILN); mesenteric lymph nodes (MLN); Peyer's patches (PP) and pancreatic lymph nodes (PLN). Black bars: STD diet. Grey bars: GF diet.

*C*ell suspensions were prepared from isolated mucosal (MLN, PP, PLN) and non-mucosal (S, ILN) lymphoid organs and stained for CD3, CD4 and Foxp3 markers, and percentages of CD4^+^Foxp3^+^ T cells was assessed within each of the studied lymphoid tissues (gating shown in Fig. 1A). No significant difference in the proportion of CD4^+^Foxp3^+^ T cells was found in the mucosal lymphoid tissues: PLN (11.6±0.11 vs. 11.5±0.06%, P = 0.27), MLN (15.1±1.99 vs. 11.9±1.14%, P = 0.29) and PP (14.5±0.10 vs. 14.2±0.19%, P = 0.36) in the BALB/c mice fed the STD compared to the GF diet. Similarly, no significant difference in percentages of CD4^+^Foxp3^+^ T cells was found in S (18.9±0.60 vs. 17.1±0.52%, P = 0.14) and ILN (11.5±0.34 vs. 11.9±0.55%, P = 0.59) between the two groups of animals (Fig. 1B). These results were confirmed by a CD3^+^CD4^+^CD25^+^ staining and analysis for CD4^+^CD25^high^ T cells (data not shown). Thus, we found no diet-induced differences in the proportion of CD4^+^Foxp3^+^ T cells, neither in mucosal lymphoid organs (MLN, PP, PLN) nor in non-mucosal lymphoid compartments (S, ILN).

### The STD diet induced a significant increase in CD4^+^IL-17^+^ (Th17) cells in PLN

**Figure 2:**
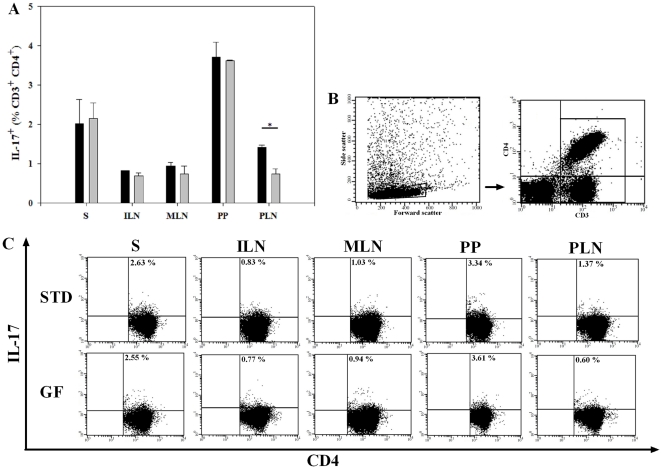
The STD diet induced a significant increase in CD4^+^IL-17^+^ (Th17) cells in PLN. (**A**) Bars represent percentages of IL-17^+^ cells gated on 100%CD3^+^CD4^+^ cells. BALB/c mice on the STD diet showed an increased proportion of CD4^+^IL-17^+^ T cells in PLN, compared to mice on the GF diet. Data are presented as mean values ± SEM of two independent experiments with 6 mice in each group. (**B**) Representative example of the lymphocyte gate and CD3^+^ gate. (**C**) Examples of dot plots showing the percentages of IL-17^+^ T cells, gated on 100% CD3^+^CD4^+^ cells. Spleen (S); inguinal lymph nodes (ILN); mesenteric lymph nodes (MLN); Peyer's patches (PP) and pancreatic lymph nodes (PLN). Black bars: STD diet. Grey bars: GF diet. * P<0.05.

Th17 cells, a population of CD4^+^ T helper cells that secretes a set of proinflammatory cytokines including IL-17, is considered a distinct cell lineage that seems to antagonize Treg development and thus promote autoimmunity [20]–[22]. To determine gluten-induced changes in the Th17 cell subset, we first gated according to the forward and side scatter parameters, followed by CD3 gating and percentages of CD4^+^IL-17^+^ cells were measured (Fig. 2 B/C). Significantly (P<0.05) higher proportion of CD4^+^ IL-17^+^ T cells was found only in PLN of BALB/c mice on the STD diet (1.4±0.05%) compared to mice fed the GF diet (0.7±0.14%)(Fig. 2A). The proportion of CD4^+^IL-17^+^ T cells in all other organs (S, ILN, MLN, PP) showed no diet-induced differences. Thus, the gluten-containing STD diet induced significantly increased proportion of Th17 cells in lymph nodes associated with pancreatic tissue (Fig. 2A/C).

### The STD diet reduced the proportion of γδ T cells

**Figure 3:**
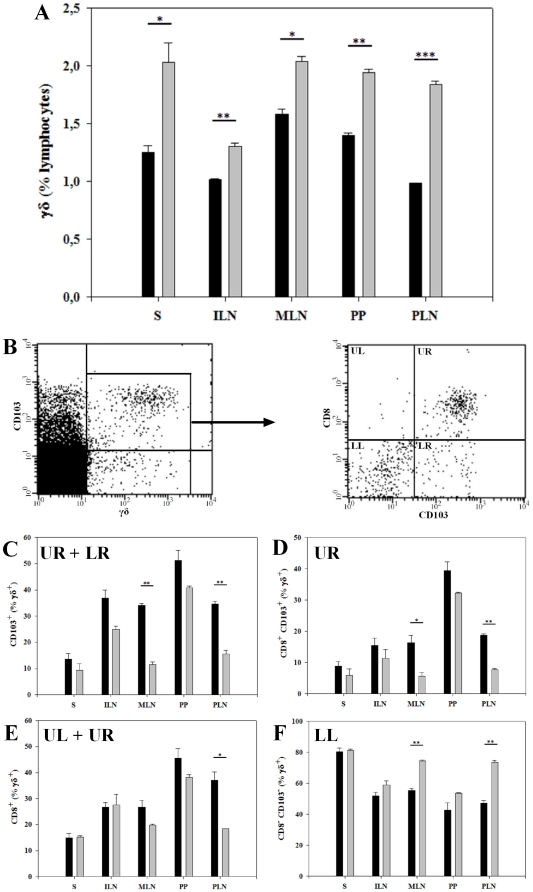
The STD diet reduced the proportion of γδ T cells. (**A**) Bars represent percentages of γδ T cells. The proportion of γδ T cells was reduced in BALB/c mice on the gluten-containing STD diet in all tested lymphoid compartments. (**B**) Representative dot plots illustrating gating of γδ T cells, and the subsequent analysis of subpopulation of γδ T cells, according to CD8 and CD103 expression. (**C**) Percentages of γδ T cells expressing CD103. Gluten induced a significant increase in CD103 expression on γδ T cells in MLN and PLN. (**D**) Percentages of CD8^+^CD103^+^ cells gated on 100% γδ^+^ cells, with an increased proportion in MLN and PLN in mice on the STD compared to the GF diet. (**E**) Percentages of γδ T cells expressing CD8. The proportion of CD8^+^ γδ T cells was higher in PLN in mice receiving the STD compared to the GF diet. (**F**) Percentages of CD8^−^CD103^−^ γδ T cells was lower in mice on the STD than on the GF diet, with a significant decrease in MLN and PLN. Barplots are mean values ± SEM of two independent experiments with 6 mice in each group. Spleen (S); inguinal lymph nodes (ILN); mesenteric lymph nodes (MLN); Peyer's patches (PP) and pancreatic lymph nodes (PLN). Black bars: STD diet. Grey bars: GF diet. * P<0.05. ** P<0.01. *** P<0.001.

Changes in the γδ T cell subset have been documented in NOD mice [23] as well as in human T1D [24]–[26], In addition γδ T cells have been shown to play an important role in induction of mucosal tolerance and prevention of T1D in the NOD mouse [27], [28]. We found a significantly lower proportion of γδ T cells in all tested lymphoid tissues in BALB/c mice fed the STD diet compared to mice receiving the GF diet, S (1.3±0.06 vs. 2.0±0.17%, P<0.05), ILN (1.0±0.01 vs. 1.3±0.03%, P<0.01), MLN (1.6±0.05 vs. 2.0±0.05%, P<0.05), PP (1.4±0.02 vs. 1.9±0.03%, P<0.01) and in PLN (1.0±0.005 vs. 1.8±0.03%, P<0.001) (Fig. 3A). Thus, the gluten-containing STD diet reduces the overall proportion of γδ T cells in both mucosal lymphoid organs (MLN, PP, PLN) and in non-mucosal lymphoid compartments (S, ILN). Next, we analysed the expression of CD8 and the mucosal homing marker CD103 within the γδ T cell subset (Fig. 3B) and observed reciprocal differences: in mice fed the STD diet there is an overall increase in γδ T cells expressing CD103, with significantly higher proportion in MLN (34.1±0.66 vs. 11.5±1.12%, P<0.01) and in PLN (34.7±1.05 vs. 15.6±1.36%, P<0.01) compared to mice receiving the GF diet (Fig. 3C). The same pattern was found when evaluating the co-expression of CD8^+^ and CD103^+^ on γδ T cells: the proportion of CD8^+^CD103^+^ γδ T cells in MLN (16.3±2.36 vs. 5.7±1.11%, P<0.05) and PLN (18.8±0.34 vs. 7.7±0.38%, P<0.01) was higher in mice receiving the STD diet compared to the GF diet (Fig. 3D). Finally, the expression of CD8 within γδ T cells was higher in PLN (37.0±3.18 vs. 18.3±0.06%, P<0.05) in mice on the STD diet vs. the GF diet (Fig. 3E). Consequently, a reversed pattern was observed in the remaining subset of CD8^−^CD103^−^ γδ T cells. Thus MLN (55.5±0.98 vs. 74.5±0.44%, P<0.01) and PLN (47.1±1.1.79 vs. 73.7±1.04%, P<0.01) of STD diet fed BALB/c mice revealed a significantly decreased proportion of CD8^−^CD103^−^ γδ T cells (Fig. 3F). In summary, there is a lower proportion of γδ T cells both in non-mucosal (S, ILN) and mucosal lymphoid organs (MLN, PP, PLN) but a higher proportion of CD8^+^γδ T cells where the majority express the mucosal homing marker CD103 in mucosal lymphoid organs (MLN, PLN) in BALB/c mice receiving the STD diet, than in mice receiving the GF diet.

### The proportion of CD4^+^T cells expressing CD45RB^high^ and CD45RB^low^ was influenced by the diet

**Figure 4:**
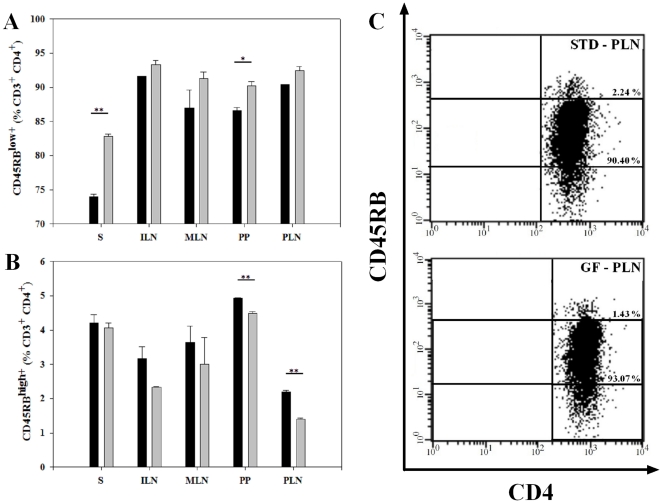
The proportion of CD4^+^T cells expressing CD45RB^high^ and CD45RB^low^ was influenced by the diet. (**A**) Bars represent percentages of CD45RB^low^ expressing cells gated on 100% CD3^+^CD4^+^ cells. There was a decreased proportion of CD4^+^ CD45RB^low^ T cells in S and PP of BALB/c mice receiving the STD diet compared to the GF diet. (**B**) Percentages of cells expressing CD45RB^high^ gated on 100% CD3^+^CD4^+^ cells. There was an increased proportion of CD4+CD45RB^high^ T cells in PP and PLN in mice receiving the STD diet. Data are presented as mean values ± SEM of two independent experiments with 6 mice in each group. (**C**) Representative dot plot of cells isolated from PLN showing the proportion of CD45RB^low^ and CD45RB^high^ CD4^+^ T cells from STD mice vs. GF mice. Spleen (S); inguinal lymph nodes (ILN); mesenteric lymph nodes (MLN); Peyer's patches (PP) and pancreatic lymph nodes (PLN). Black bars: STD diet. Grey bars: GF diet. * P<0.05. ** P<0.01.

The CD45RB antigen is highly expressed on various subsets of lymphocytes. The expression of CD45RB on CD4^+^ T cells distinguishes naïve (CD45RB^high^) from activated or memory (CD45RB^low^) cells. Furthermore, the expression of CD45RB is associated with distinct immunological functions of the T cell [29], [30]. We found that BALB/c mice on the STD diet displayed a significant lower proportion of CD4^+^CD45RB^low^ T cells in S (73.9±0.35 vs. 82.8±0.40%, P<0.01) and in PP (86.6±0.49 vs. 90.2±0.61%, P<0.05), than mice on the GF diet (Fig. 4A). The reciprocal CD4^+^CD45RB^high^ T cell population was increased in mice on the STD compared to the GF diet, with a significant increase in PP (4.9±0.01 vs. 4.5±0.05%, P<0.01) and PLN (2.2±0.05 vs. 1.4±0.03%, P<0.01) (Fig. 4B). A representative example of the CD45RB FACS analysis and the differences in the proportion of CD45RB expression in PLN is shown in Fig. 4C. In conclusion, mice receiving the gluten-containing STD diet displayed a significantly lower proportion of CD4^+^CD45RB^low^ T cells in S and PP, and a higher percentage of CD4^+^CD45RB^high^ T cells in PP and PLN.

### Fewer CD4^+^ T cells in PP expressed CD62L in mice receiving the STD diet

**Figure 5:**
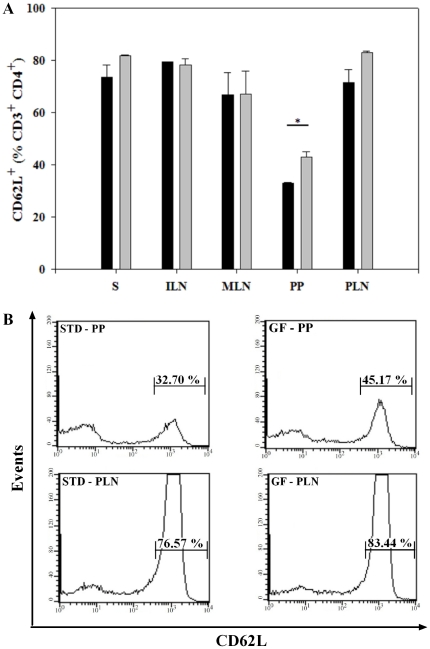
Fewer CD4^+^ T cells in PP expressed CD62L in mice receiving the STD diet. (**A**) Bars represent percentages of CD62L^+^ cells gated on 100% CD3^+^CD4^+^ cells. In BALB/C mice receiving the STD diet, compared to mice receiving the GF diet, there was a decreased proportion of CD4^+^CD62L^+^ T cells in PP. Data are presented as mean values ± SEM of two independent experiments with 6 mice in each group. (**B**) Representative histograms from PP and PLN showing the differences in CD4^+^CD62L^+^ T cells. Spleen (S); inguinal lymph nodes (ILN); mesenteric lymph nodes (MLN); Peyer's patches (PP) and pancreatic lymph nodes (PLN).Black bars: STD diet. Grey bars: GF diet. * P<0.05.

CD62L (L-selectin) is a member of the selectin adhesion molecule family, and is required for leukocyte entry from circulation into secondary lymphoid tissues such as peripheral lymph nodes [31]. When studying an effect of the STD gluten-containing diet on CD62L expression within the CD4+ T cells (Fig. 5), we found no substantial differences in non-mucosal lymphoid compartments (S, ILN) as well as in MLN and PLN associated with the mucosal lymphoid tissue. However, there were significantly fewer CD4^+^CD62L^+^ T cells in mice receiving the STD diet compared to the GF diet in PP (32.9±0.26 vs. 42.9±2.32%, P<0.05)(Fig. 5A). A representative example of isolated PP is shown in Fig. 5B. Although not significant, a decreased percentage of CD62L^+^CD4^+^ T cells was also noticed in PLN in mice receiving the STD diet. In a representative example we found 76.57% compared to 83.44%. In conclusion, there were fewer CD4^+^ T cells expressing the CD62L homing marker in PP of BALB/c mice fed the STD diet than in mice on the GF diet but no differences were found in CD62L expression on CD4^+^ T cells in all other lymphoid compartments (S, ILN, MLN, PLN).

### Increase in CD103^+^ T cells in S and PLN in mice receiving the STD diet

**Figure 6:**
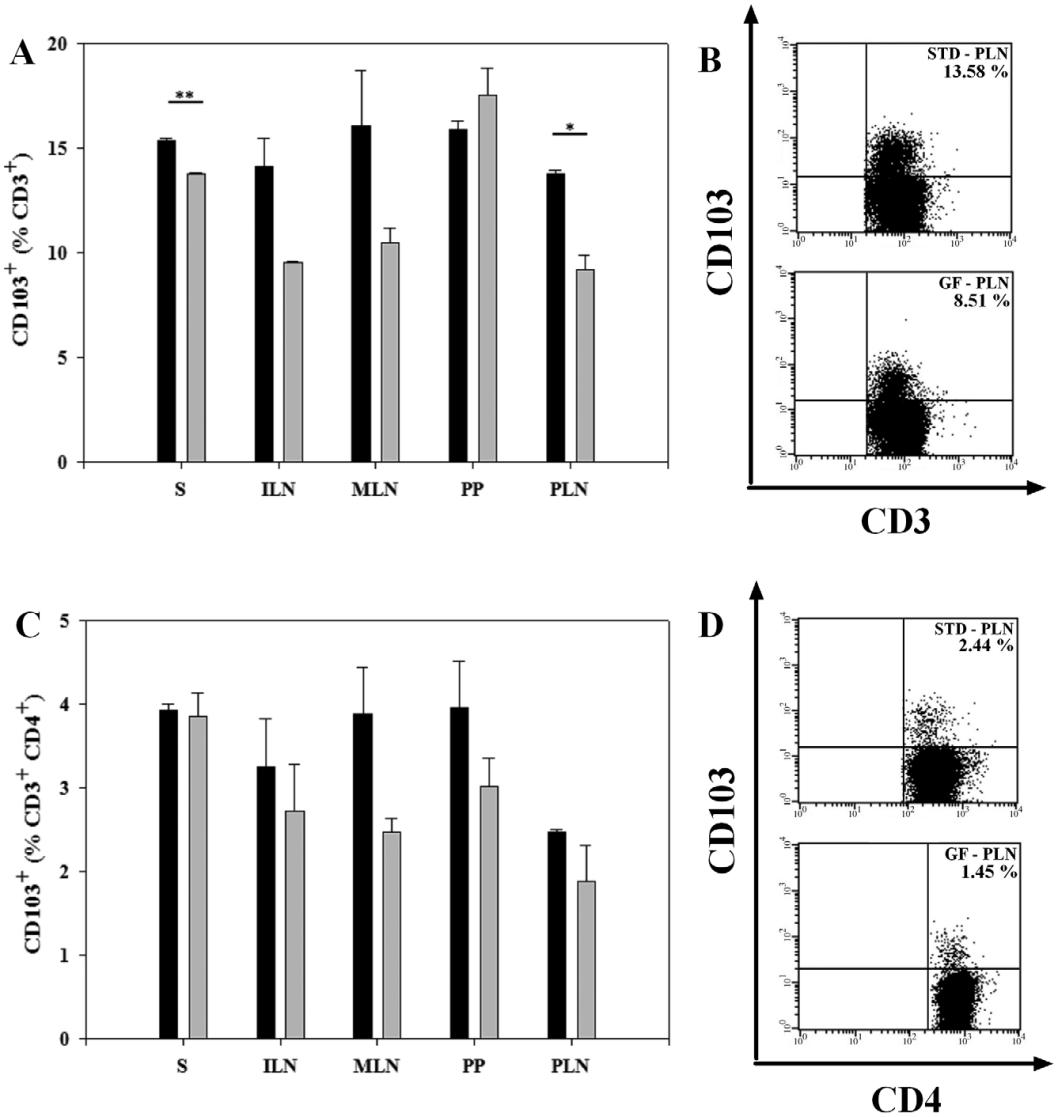
Increase in CD103^+^ T cells in S and PLN in mice receiving the STD diet. (**A**) Bars represent the proportion of CD103^+^ cells, gated on 100% CD3^+^ cells. There was an increased proportion of CD103 expression in S and PLN in BALB/c mice receiving the STD diet. (**B**) Representative dot plot from PLN showing the differences in CD103^+^ T cells between the two diets. (**C**) Bars showing the percentages of CD103^+^ cells gated on 100% CD3^+^CD4^+^ lymphocytes. (**D**) Representative dot plots of CD4^+^CD103^+^ T cells from isolated PLN. Barplots are mean values ± SEM of two independent experiments with 6 mice in each group. Spleen (S); inguinal lymph nodes (ILN); mesenteric lymph nodes (MLN); Peyer's patches (PP) and pancreatic lymph nodes (PLN). Black bars: STD diet. Grey bars: GF diet. * P<0.05. ** P<0.01.

The integrin CD103 (αEβ7 integrin) is responsible for an efficient homing and retention of lymphocytes to gut-associated lymphoid tissue, gut epithelium and lamina propria [32]. To determine diet-induced differences in CD103 expression on T cells, we first assessed differences within the CD3^+^ gated cells. We found a significantly higher proportion of CD103^+^ T cells in mice on the STD diet vs. the GF diet, in S (15.4±0.11 vs. 13.8±0.06%, P<0.01) and in PLN (13.8±0.20 vs. 9.21±0.70%, P<0.05) (Fig. 6A). A representative staining of CD3^+^CD103^+^ cells isolated from PLN is shown in Fig. 6B. However, when gating on CD3^+^CD4^+^ T cells we found no significant diet-induced differences in the CD103 expression (Fig. 6C/D). CD103 positive T cells are thus found to be expanded in S and PLN of BALB/c mice receiving the STD diet, compared to the GF diet, but this pattern is not present within the CD4^+^ T cells.

### No diet-induced difference was found in the proportion of CD49b^+^ T cells and CD49b^+^ non-T cells

**Figure 7:**
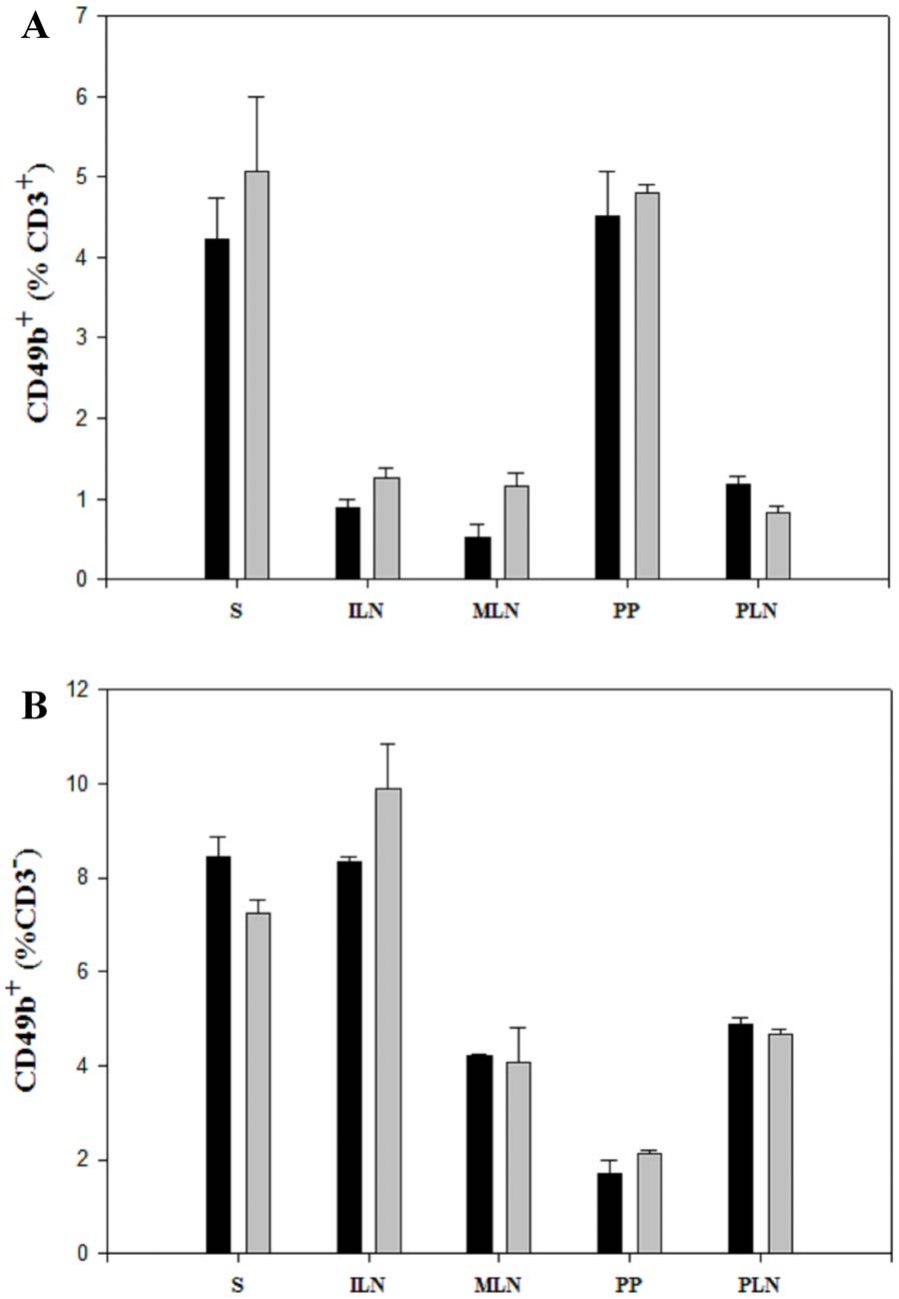
No diet-induced difference was found in the proportion of CD3^+^CD49b^+^ (NKT cells) or CD3^−^CD49b^+^ (NK cells). (**A**) Bars represent the proportion of CD49b^+^ cells, gated on 100% CD3^+^ cells. No diet induced differences was found. (**B**) Bars showing the percentages of CD3^−^CD49b^+^ cells, with no significant differences between the two diets. Barplots are mean values ± SEM of two independent experiments with 6 mice in each group. Spleen (S); inguinal lymph nodes (ILN); mesenteric lymph nodes (MLN); Peyer's patches (PP) and pancreatic lymph nodes (PLN). Black bars: STD diet. Grey bars: GF diet.

Because BALC/c mice do not express the NK1.1 marker, we have used anti-CD49b (clone DX5) pan-NK mAb that was shown to almost entirely overlap with the NK1.1 staining in C57Bl/6 mice as an NK cell marker [33]. We assessed its expression within the CD3^+^ and the CD3^−^ cells. No statistically significant difference in proportions of CD3^+^CD49b^+^ or CD3^−^CD49b^+^ cells were found in neither S, ILN, MLN, PP, PLN of BALB/c mice fed the STD versus GF diets (Fig. 7A/B). Although not significant a tendency towards a lower level of CD3^+^CD49b^+^ (NKT cells) were found in mice fed the STD versus the GF diet (Fig. 7A).

## Discussion

T1D is an autoimmune disease that is characterized by dysregulated T cell activation and diminished regulatory T cell function [34], [35]. Gluten intake has been associated with the development of T1D in both animal models of T1D [1], [3] and in humans [4], [5], but the effect of dietary gluten on T cell subsets associated with regulatory function has not been well characterized. In order to assess the effect of dietary gluten on various T cell subsets, experiments were carried out in healthy immunocompetent BALB/c mice, as NOD mice are known to be lymphophenic [28] as well as they display other immune alterations [36].

**Figure 8:**
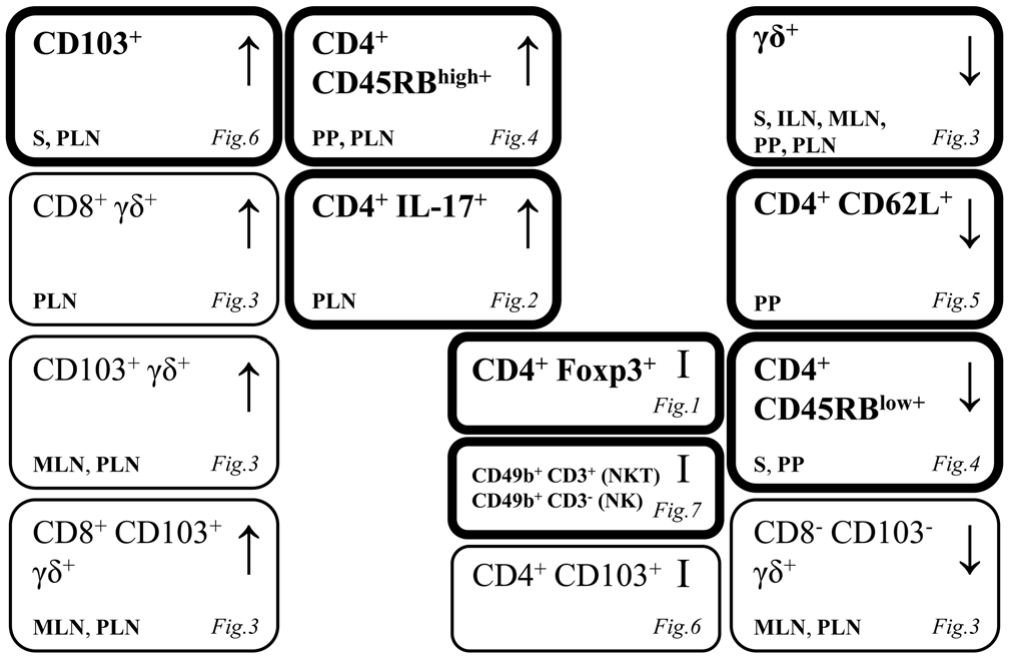
Summary of changes in T cells (CD3^+^) subsets and cellular markers in BALB/c mice receiving the gluten-containing STD diet compared to the GF diet. Spleen (S); inguinal lymph nodes (ILN); mesenteric lymph nodes (MLN); Peyer's patches (PP) and pancreatic lymph nodes (PLN). Significant diet-induced changes within specific lymphoid compartment are indicated in each box. ↑: Increased proportion in animals receiving the gluten-containing STD diet. ↓: Decreased proportion in animals receiving the gluten-containing STD diet. I: No diet-induced changes. Boxes with thin line: Subsets of T cells expressing a specific cellular marker e.g. γδ T cells expressing CD103.

Collectively, our data show that a gluten-containing STD diet decreases the proportion of γδ T cells in all lymphoid compartments studied, but reciprocal differences was detected in CD8^+^ and CD103^+^ γδ T cells in mucosal lymphoid compartments. This is the most consistent change among the T cell subsets studied according to gluten influenced changes. However we also found substantially decreased proportion of CD62L^+^CD4^+^ T cells in PP and of CD4^+^CD45RB^low+^ T cells in S and PP associated with the STD diet. Furthermore, mice fed the STD diet showed increased proportion of CD4^+^CD45RB^high+^ in PP and PLN and of CD103^+^ T cells in S and PLN. Interestingly, no diet-induced changes were found in CD4^+^Foxp3^+^ T cells, CD3^+^CD49b^+^ (NKT cells) or CD3^−^CD49b^+^ (NK cells). The Th17 cell population, associated with the development of autoimmunity [37], [38], was substantially increased in PLN of mice fed the STD diet. A summary of the changes in T cells (CD3^+^) subsets and cellular markers in BALB/c mice receiving the gluten-containing STD diet compared to the GF diet is given in Fig. 8.

Evidence derived from both human and animal studies suggests that a deficiency in either the frequency or the function of Tregs could be associated with T1D development [34]. Foxp3-expressing Tregs are a constitutively occurring T cell subpopulation, whose deficiency is associated with development of autoimmunity, and which controls adaptive immune responses both in health and disease [39]. We found no diet-induced changes in the proportion of Foxp3^+^ Tregs in neither mucosal lymphoid tissue (MLN, PP, PLN) nor in non-mucosal lymphoid compartments (S, ILN). Previously, one study reported that gluten intake reduces the proportion of Foxp3^+^ T cells in PP of BALB/c mice [40]. The discrepancy between the studies could be due to differences in the age at which the BALB/c mice were introduced to the gluten-free diet or whether the mice were exposed to gluten during lactation. Other studies do, however, find that the proportion of Foxp3^+^ Tregs is not influenced by gluten intake as in human CD patients [41] or in T1D patients or in individuals at varying degrees of risk for T1D [42]. Thus it seems that the frequency of Foxp3^+^ Tregs is not related to disease development or prevention but a defective regulatory function in Foxp3^+^ Tregs could still play a role in susceptibility to the disease. This view is supported by studies showing defective suppressor function in CD4^+^CD25^+^ T cells from T1D patients [43], [44] and by studies showing that Foxp3 mRNA expression (relative to the number of Foxp3^+^ Tregs) is reduced in patients with potential CD or T1D [45], [46]. The authors suggest that this could indicate that the maintenance of oral tolerance to gluten is not related to a higher proportion of Foxp3^+^ T cells, but is related to the up-regulation of Foxp3 transcripts [46]. Therefore it seems that functional changes in Foxp3^+^ Tregs, rather than their frequency might be involved in the susceptibility for developing T1D.

Interestingly, we found that the gluten-containing STD diet specifically raised the proportion of Th17 cell in PLN. The Th17 subsets are considered to be a key mediator of distinct autoimmune diseases, as rheumatoid arthritis [47], multiple sclerosis [48], inflammatory bowel disease [49], and CD [50], [51]. Recently Th17 cells have been described to be involved in T1D, both in animal models [38] and in humans [37]. The exact role of Th17 cells in the diabetogenic process remains to be fully determined. However, it has been shown that the insulitis lesions in the NOD mice contain high levels of IL-17 transcript [52]. Moreover, inhibition of Th17 cells with neutralising anti-IL-17 antibodies or with recombinant IL-25 prevents diabetes in NOD mice when treatment is started at 10 weeks but not 5 weeks of age [53], illustrating the importance of this cell population in the pathogenesis of T1D as well as the role of different time windows required for T1D preventive interventions. To our knowledge, gluten-induced Th17 priming in pancreatic lymph nodes has not previously been described. This finding thus points towards possible mechanisms by which dietary gluten may have an etiological role in T1D.

Several lines of evidence suggest a role for γδ T cells in T1D [24]–[26]. Their role in the pathogenesis seems to be complex, with indications of their involvement both in regulatory and pathogenic pathways. NOD mice display an increased proportion of γδ T cells at onset of diabetes [23], and in BB rats the development of γδ T cells is inhibited due to the *lyp* mutation, which is a marker of susceptibility to diabetes [54]. Recently, insulin-specific γδ T cells were found in NOD mice, and the authors suggest that these insulin-specific γδ T cells are involved in the auto-immune response leading to T1D [55]. We found a significantly lower proportion of γδ T cells both in non-mucosal and mucosal lymphoid organs in mice receiving the STD diet, compared to the GF diet. This is the most consistent change detected among the T cell subsets, in respect to the two diets tested. However, a higher proportion of CD8^+^ γδ T cells and γδ T cells expressing the mucosal homing marker CD103 were found in mice on the STD diet vs. the GF diet. Although, the exact role of γδ T cells is remains to be further studied, several findings have documented an importance of γδ T cells in induction of tolerance. Thus, nasal exposure to insulin induces CD8^+^γδ T cells capable of suppressing diabetes development in mice [27], anti-γδ antibody disrupts oral mucosal tolerance to ovalbumin in mice [56], and oral administration of antigen does not induce tolerance in TCRδ-knockout mice [57]. Moreover, it have been shown that intraepithelial CD8^+^γδ T cells prevent diabetes, and intraepithelial γδ T cells are required for induction of regulatory CD4^+^CD25^+^ T cells by oral insulin, in the neonatal thymectomy NOD mouse model of T1D [28]. These data suggest that oral tolerance to antigens is induced and actively maintained by mechanisms involving γδ T cells. Overall, the intake of dietary gluten reduces the proportion of γδ T cells in both non-mucosal and mucosal lymphoid organs, but it increases the proportion of potentially regulatory CD8^+^ γδ T cells expressing the intestinal homing marker CD103 in mucosal lymphoid organs. To support these findings, we determined diet-induced differences in the expression of CD103. We found an increase in the proportion of CD103^+^ T cells in S and PLN of BALB/c mice receiving the gluten-containing STD diet. The CD103 integrin (αEβ7 integrin) is important for homing of T cells to the intestinal compartment, as it mediates adhesion to epithelial cells due to its binding to E-cadhedrin, expressed selectively on epithelial cells [58]. Our data indicate that gluten leads to an immune activation within the intestinal compartment that causes upregulation of CD103 on lymphocytes.

Several T cell subsets with regulatory properties have been demonstrated to prevent diabetes development in the NOD mouse model. Apart from the regulatory role of γδ T cells in T1D [27], [28] CD4^+^CD62L^+^ T cells have been shown to inhibit development of the disease in NOD mice [59], [60] Here we show a decreased proportion of CD4^+^CD62L^+^ T cells in PP and a similar tendency in PLN of BALB/c mice fed the gluten-containing STD diet compared to the GF diet. Since expression of CD62L is critical for leukocyte trafficking into secondary lymphoid organs [31], the diabetes-permissive STD diet may be associated with lower proportion of naïve CD4^+^ T cells migrating to mucosal compartments such as PP or the PLN. The increased proportion of CD62L expression in PP and a similar tendency in PLN in mice fed the diabetes-preventive GF diet is also interesting in respect to the observation that Tregs expressing high levels of CD62L posses stronger immunosuppressive potential [61].

Transfer of CD4^+^CD45RB^high^ expressing T cells into an immunodeficient mouse induces chronic intestinal inflammation [62] and this population of cells is conventionally used to induce inflammatory bowel disease in mice [63], illustrating their pathogenic potential. Co-transfer of lymphocytes expressing CD4^+^CD45RB^low^, on the other hand, prevents disease development [64]. Gluten-induced changes in CD45RB expression on CD4^+^ T cells could be important for T1D development since subpopulations of CD4^+^ T cells expressing high or low levels of CD45RB, have different cytokine profiles and mediate distinct immunological functions [65]. CD45RB^low^ T cells were reported to have a regulatory cytokine profile with IL-10 and IL-4 production [30], opposite to CD45RB^high^ expressing cells, producing more proinflammatory cytokines as IFNγ [29] and TNFα [30]. We found an increase of CD4^+^CD45RB^high^ T cells in PP and PLN, while the CD4^+^CD45RB^low^ T cell population was decreased in S and PP, in mice fed the STD diet. Gluten may therefore increases a subset of CD4^+^ T cells, expressing CD45RB^high^, that may induce inflammation in the intestine, perhaps due to their secretion of proinflammatory cytokines. In this respect, it is interesting that the proportion of CD4^+^CD45RB^high^ T cells was increased only in mucosal compartments (PP, PLN).

In summary, our present study reveals that the gluten-containing STD diet, that allows high diabetes penetrance in spontaneous, animal models of T1D, significantly decreases the proportion of different potentially regulatory T cell populations, especially γδ T cells and CD4^+^CD62L^+^, but not CD4+Foxp3^+^ T cells. Moreover, the STD diet modifies the proportions of CD4^+^CD45RB^low+^ and CD4^+^CD45RB^high+^ T cells and leads to an increased proportion of proinflammatory Th17 cells in pancreas draining lymph nodes. Interestingly, many of the described changes were localized specifically within the mucosal compartment (MLN, PP) and PLN. Thus, our data show the effect of dietary gluten on the gut-associated immune system and the potential, etiological role of gluten in T1D. We suggest that failure to develop proper regulatory immune responses to environmental stimuli, such as dietary gluten, may lead to a higher incidence of T1D in genetically predisposed individuals.
